# Comparative Phenotypic Analysis of *Anabaena* sp. PCC 7120 Mutants of Porinlike Genes

**DOI:** 10.4014/jmb.2103.03009

**Published:** 2021-04-06

**Authors:** Hannah Schätzle, Eva-Maria Brouwer, Elisa Liebhart, Mara Stevanovic, Enrico Schleiff

**Affiliations:** 1 Institute for Molecular Biosciences, Goethe University, Frankfurt am Main, Germany; 2 FIERCE, Goethe University, Frankfurt am Main, Germany; 3 Buchmann Institute for Molecular Life Sciences, Goethe University, Frankfurt am Main, Germany; 4 Frankfurt Institute of Advanced Studies, Frankfurt am Main, Germany

**Keywords:** Cyanobacteria, β-barrel proteins, Omp85 function, outer membrane biogenesis, porins

## Abstract

Porins are essential for the viability of Gram-negative bacteria. They ensure the uptake of nutrients, can be involved in the maintenance of outer membrane integrity and define the antibiotic or drug resistance of organisms. The function and structure of porins in proteobacteria is well described, while their function in photoautotrophic cyanobacteria has not been systematically explored. We compared the domain architecture of nine putative porins in the filamentous cyanobacterium *Anabaena* sp. PCC 7120 and analyzed the seven candidates with predicted OprB-domain. Single recombinant mutants of the seven genes were created and their growth capacity under different conditions was analyzed. Most of the putative porins seem to be involved in the transport of salt and copper, as respective mutants were resistant to elevated concentrations of these substances. In turn, only the mutant of *alr2231* was less sensitive to elevated zinc concentrations, while mutants of *alr0834*, *alr4741* and *all4499* were resistant to high manganese concentrations. Notably the mutant of *alr4550* shows a high sensitivity against harmful compounds, which is indicative for a function related to the maintenance of outer membrane integrity. Moreover, the mutant of *all5191* exhibited a phenotype which suggests either a higher nitrate demand or an inefficient nitrogen fixation. The dependency of porin membrane insertion on Omp85 proteins was tested exemplarily for Alr4550, and an enhanced aggregation of Alr4550 was observed in two *omp85* mutants. The comparative analysis of porin mutants suggests that the proteins in parts perform distinct functions related to envelope integrity and solute uptake.

## Introduction

Cyanobacteria are Gram-negative bacteria, as they possess an outer membrane (OM) that acts as diffusion barrier, a peptidoglycan mesh (PG) and a plasma membrane (PM). The cyanobacterial envelope in certain aspects differs from that of other Gram-negative heterotrophs [1]. Exemplarily the PG layer in filamentous *Anabaena* sp. strain PCC 7120 (*Anabaena* sp.) is approximately 14 nm thick, and the distance between PM and OM is about 45 nm [2]. Here, the outer membrane continuously surrounds the whole filament by not penetrating into the septum area between two cells [2]. Compared to that the PG of *E. coli* is approximately 6 nm thick and the distance between PM and OM is in the range of 20 nm [3]. The denoted dimensions of the cell envelope might be even disparate within certain cyanobacterial species, as some multicellular cyanobacteria species possess different cell types with distinct cell envelope properties.

Membrane proteins regulate transport processes across membranes and define the susceptibility of an organism against harmful compounds and antibiotics. A protein class that is highly abundant in the OM are porins. Porins are membrane embedded β-barrel proteins that allow diffusion of substrates with low or high specificities (reviewed *e.g.* in [4, 5]). Their functions are not only related to diffusion and solute transport, but porins are also crucial for outer membrane integrity including antibiotic resistance [6] or pathogenesis [4]. Porins that lack specificity are termed general or non-specific porins. They typically consist of 16β-strands and facilitate diffusion of small hydrophilic compounds [7]. In addition, substrate specific porins exist, typically composed of 18β-strands [8]. Porins with less than 16β-strands are described as well, for example the monomeric OmpG from *Escherichia coli* is composed of 14β-strands [9]. Although monomeric porins have been reported, porins most often occur as trimers [5]. Porins and other outer membrane β-barrel proteins are integrated into the OM by the β-barrel assembly machinery [10]. An Omp85 protein constitutes the main pore of this protein complex. The N-terminal part of the Omp85 protein bears polypeptide transport-associated domains (POTRA) that recognize the non-folded membrane proteins. Moreover, the POTRA domains interact with periplasmic chaperones or other proteins involved in the insertion and folding process [11].

The freshwater cyanobacterium *Anabaena* sp. is a model organism with regard to bacterial cell differentiation, as in the absence of a combined nitrogen source specialized cells called heterocysts are formed. Heterocysts are morphologically distinct from vegetative cells, including the structure of the cell envelope [12]. They are surrounded by a polysaccharide layer that mechanically protects the underneath glycolipid layer. The prevalence of certain proteins in the outer membrane only mildly differs between vegetative cells and heterocysts [13]. Information on cyanobacterial porins is rather limited compared to proteobacteria. Cyanobacterial porins were found to be relatively large with about 30−50 kDa per monomer, whereas most proteopbacterial porin monomers have a molecular weight lower than 40 kDa [14-16]. The enlargement results from the presence of an additional cyanobacteria specific N-terminal domain [17]. This domain is related to the conserved surface layer homology (SLH) domain, which is involved in targeting and linking proteins to cell-wall associated components [18-21].

Examinations of SomA and SomB, two out of six porin-like proteins in the unicellular cyanobacterium *Synechocystis* sp. strain PCC 6803 (*Synechocystis* sp.), indicated that the permeability of organic compounds is relatively low (0.4 and 0.9 nS) [17]. Hence, it is hypothesized that *Synechocystis* sp. lacks classical porins. The major OM proteins did not allow diffusion of organic substances, whereas inorganic molecules could penetrate the pores [22]. Thus the overall permeability of the *Synechocystis* sp. OM was around 20-fold lower than that of *E. coli*. The authors claim that this might be a consequence of the photoautotrophic lifestyle of *Synechocystis* sp., which renders an import of sugars dispensable. Cyanobacteria that are living in symbiosis with plants seem to represent an exception, as the photosynthetic activity in those organisms is often diminished. In a symbiotic species of the cyanobacterium *Nostoc punctiforme* the sugar-specific porin OprB was found to be required for proper uptake of sugars [23]. Notably an OprB-type porin together with the Omp85 protein and LptD, a protein that is involved in the transfer of lipid A, was found to be globally conserved among cyanobacterial OM proteins [24]. However, there is no indication whether those porins are truly carbohydrate-selective or not. Recently it was described that in *Synechocystis* sp. the porin Slr1908 mediates the transport of inorganic iron [25]. Slr1908-like sequences were identified in many cyanobacterial species, indicating a porin-dependent iron uptake in cyanobacteria

Nine porin-like genes were assigned based on sequence alignments and amino acid sequence properties in the genome of *Anabaena* sp. [26-28], and previous analyses showed that porins might be involved in ethidium bromide and presumably erythromycin uptake in *Anabaena* sp. [29]. The central Omp85 proteins of *Anabaena* sp. are encoded by *alr0075*, *alr2269* and *alr4893*. Proteomic analyses revealed that Alr2269 is the most abundant among the three Omp85 proteins, whereas Alr4550, All4499 and Alr3608 are the most abundant among the putative porins [28].

Here, we comparatively characterize the seven genes coding porin-like proteins in *Anabaena* sp. by analyzing the transcript abundance of the genes under standard and starvation conditions. Although the function of these protein has not been biochemically characterized, for simplification and easier reading we subsequently refer to the proteins as “porins” based on the sequence and motif similarity to characterized proteins in proteobacteria. Moreover, mutants of the single porin-like genes were phenotypically analyzed in presence of high metal, salt and drug concentrations. The results indicate that the highly abundant porin Alr4550 plays an important structural role in *Anabaena* sp., since the corresponding mutant was strongly defected in OM integrity. Further, a function in the transport of manganese, zinc or cobalt is suggested for specific porins. Interestingly a mutant of *all5191* was altered in growth under diazotrophic conditions, which suggests an insufficient nitrogen fixation capacity or an enhanced nitrogen demand of the strain. Moreover, we show that Alr2269 and Alr4893 affect the membrane integration of the porin Alr4550, which was analyzed as model substrate. This implies a functional diversification of the three Omp85 proteins.

## Materials and Methods

### Bioinformatics

Sequences and sequence information were extracted from NCBI database [30]. Logo plots were created using the WebLogo online tool (http://weblogo.berkeley.edu/logo.cgi, [31]). The sequence alignment for determination of the sequence identities was performed with Clustal Omega [32, 33].

### Generation of *Anabaena* sp. PCC 7120 Mutants

An internal fragment of the gene of interest was amplified by PCR with gene specific oligonucleotides (Table S1). BglII-sites were introduced at 5’ and 3’-ends of the fragment. This fragment was inserted into BamHI-digested pCSV3 carrying a CS.3 cassette (kind gift from Prof. E. Flores, [34, 35]) yielding the final plasmid for conjugation (Table S2). Single-recombinant insertion mutants of *Anabaena* sp. were created with the triparental mating method [36-38] utilizing *E. coli* strains HB101 (gets transformed with the plasmid of interest) and ED8654 (carries the conjugative plasmid). In short the two *E. coli* strains were mixed with *Anabaena* sp. wild type, and the plasmid of interest bearing a homolog region to the gene of interest is transferred to *Anabaena* sp. by conjugation. A single recombination event happens where the whole plasmid bearing the homolog region gets integrated into the genome, resulting in cells that are resistant towards spectinomycin and streptomycin.

The genotype of the exconjugants was tested by PCR utilizing oligonucleotides that anneal outside of the internal homologous fragment in combination with a vector-specific oligonucleotide (Table S1). The mutant strains are listed in Table S3.

### Phenotypic Analysis of the Porin Mutants

*Anabaena* sp. strains were stored on BG11 plates [39] with 1% (w/v) bacto-agar (BD Biosciences) until use. For expression analysis by qRT-PCR *Anabaena* sp. wild type and mutants were grown in buffered liquid YBG11 medium [40]. In case of mutants the medium was supplemented with 5 μg ml^-1^ spectinomycin dihydrochloride pentahydrate (Duchefa Biochemie) and streptomycin sulfate (Roth).

For the phenotyping on plates, cultures were washed and suspended at a final concentration OD_750nm_=1. 5 μl of the non-diluted suspension and of a 1:10 dilution was spotted onto YBG11 plates containing given amounts of supplements or on YBG11_0_ plates (YBG11_0_: YBG11 medium without NaNO_3_). Plates were incubated under constant illumination (70 μmol photons m^-2^ s^-1^) at 28-29°C for 7 days. Each spotting assay was repeated with independent cultures at least three times. Representative images are shown in the results. All strains depicted in one line were grown on the same plate.

### Microscopy

Light microscope images were taken with Olympus CKX41 using a 40x objective and a Thorlabs DCC1645C-HQ camera. Heterocysts were stained with alcian blue to improve visibility. *Anabaena* sp. suspension was mixed in a 1:1 ratio with the alcian blue staining solution (0.5% alcian blue in 50% ethanol, [41]) and incubated for 5 min. After three washing steps with YBG11_0_ medium (centrifugation at 1500 g, 5 min) filaments were inspected under the microscope.

### RNA Isolation and qRT-PCR Analysis

RNA isolation, cDNA synthesis and qRT-PCR with the corresponding oligonucleotides (Table S1) was performed as previously described [42]. Three independent wild-type and mutant cultures grown for 5 days were used for RNA isolation, while for starvation experiments three week-old cultures were used. qRT-PCR was performed on cDNA from three biological replicates. The Ct values for the genes were normalized to Ct values of the *rnpB* transcript in the corresponding sample, yielding the ΔCt. For the calculation of the ΔΔCt-value the ΔCt for each sample was normalized to the ΔCt of wild type grown under control conditions [43].

### Membrane Isolation and Rate Zonal Centrifugation

OM from *Anabaena* sp. was isolated as described [30] with slight modifications. *Anabaena* sp. cultures were grown in BG11 to exponential growth phase, 500 ml were harvested and washed with 5 mM 4-(2-hydroxyethyl)-1-piperazineethanesulfonic acid (HEPES), pH 7.6 supplemented with 1 mM phenylmethylsulfonyl fluoride (PMSF). Cell lysis and harvesting of membrane fractions was performed as described [30]. The membrane fraction was resuspended in 30 % sucrose containing 20 mM HEPES, pH 7.6 and 0.2 mM PMSF. It was layered on top of a 55% (w/v) sucrose cushion. The OM was sedimented by centrifugation (130,000 ×*g*, 16 h, 4°C). The OM pellet was washed with 20 mM HEPES, pH 7.6 supplemented with 0.2 mM PMSF and collected by centrifugation (130,000 ×*g*, 1 h, 4°C). Membranes were resuspended in 20 mM HEPES, pH 7.6 with 0.2 mM PMSF and stored at -80°C.

100 μg protein of the membrane fraction was loaded on top of a linear sucrose gradient (10 to 70%; w/v) and centrifuged (100,000 ×*g*, 16 h, 4°C). The gradient was fractionated into ten fractions of 1 ml. Proteins were precipitated with 0.02% (w/v) Na-deoxycholate and 15% (v/v) trichloroacetic acid. Precipitates were resolved in 6-fold SDS-Urea loading buffer containing 200 mM Tris HCl pH 6.8; 8M Urea; 0.1 mM ethylenediaminetetraacetic acid (EDTA); 5% sodium dodecyl sulfate (SDS) and 0.03% bromophenol blue and subjected to SDS-PAGE followed by immunoblotting. Blotting membranes were stained with direct blue 71 (DB71) as described [44].

For total protein extract 1 ml of exponential phase culture was harvested by centrifugation (10,000 ×*g*, 5 min) and the pellet was resuspended in 6-fold SDS-Urea loading buffer (recipe given above). Samples were incubated at 42°C for 5 min and centrifuged afterwards to pellet insoluble cell debris, the supernatant was used for SDS PAGE.

Antibodies against the peptides specific for Alr4450 were generated by Peptide Specialty Laboratories (Germany) and previously described [45], the following peptides were used: Peptide 1: ASGQGLQTFQVGSTGNNC, Peptide 2: PITEDTKVIDQVNRYSNEGKGNAQ. Antibodies against Alr2269 and Tic22 have also been previously described [30,46].

### Inductively Coupled Plasma Mass Spectrometry (ICP-MS)

The intracellular metal concentration of *Anabaena* sp. wild type grown for seven days in YBG11 medium was analysed. The procedure was adapted [47] with modifications. The cells were sedimented by centrifugation (3,000 g, 10 min) and subsequently washed twice with 20 mM 2-(N-morpholino)ethanesulfonic acid (pH 5) and 10 mM EDTA. The final pellet was resuspended in double-distilled water (ddH_2_O). For normalization the OD_750_ was determined and the cells were counted using a Helber bacteria counting chamber (Hawksley). From here on experiments were conducted in a metal clean laboratory. 1 ml of each sample was incubated at 120°C in 7 M HNO_3_ overnight until dryness. Before measurement, the samples were resolved in 5% HNO_3_. As controls, samples of ddH_2_O and cultivation medium were analysed. Glassware used during the experiment was incubated in 4% HNO_3_ overnight prior to use.

## Results

### Seven Proteins in *Anabaena* sp. Contain a Porin-like Domain Architecture

In previous studies, the nine putative porin-candidates *alr0834*, *alr2231*, *all4499*, *alr4550*, *alr4741*, *all5191*, *all7614*, *alr3917* and *alr3608* were identified in the *Anabaena* sp. genome [30, 48]. In addition, All3289 and Alr5049 were assigned as OmpA-like proteins [48]. OmpA-type porins are thought to perform structural functions related to membrane integrity [8, 49]. However, *all3289* codes for a protein with 1289 amino acids. The C-terminal region might form an OM anchor for the large soluble domain that contains characteristics of glycoproteins. Hence, we suggest that this protein should not be considered as typical OmpA. In turn, *alr5049* codes for a protein with 169 amino acids lacking the characteristic domains of an OmpA [48]. Thus, based on the small size, the absence of domains characteristic for OmpA proteins and considering that the outer membrane localization can be confirmed in future, the protein might represent a functional OmpX [50].

**Fig. 1:**
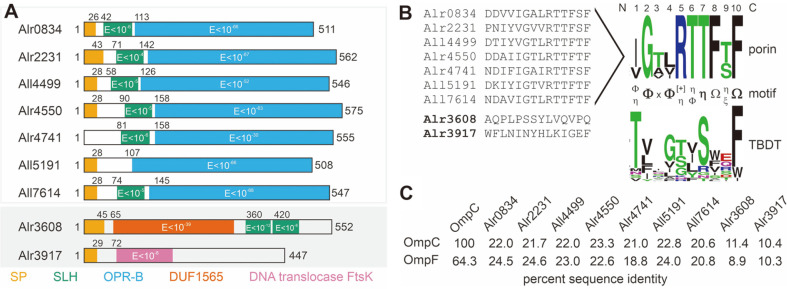
Domain structure of the putative porins in *Anabaena* sp. PCC 7120. (**A**) Shown is the domain structure of the nine predicted porins highlighting the signal peptide (SP, orange; predicted with Signal P 5.0), the SLH domain (green, extracted from the CCD server, the E-value for prediction is given), the OPR-B domain (blue), the Domain of unknown function 1565 (red) and the provisional DNA translocase FtsK domain fragment (purple). (**B**) The last 14 C-terminal amino acids of the predicted porins are shown in the alignment. The LOGO plot for the seven proteins with OPR-B domain and for the predicted 22 TBDTs is shown. The motif of the last β-strand found in the putative porins and the TBDTs is highlighted with the following nomenclature: Ω aromatic amino acids, η hydroxylated amino acids, Φ hydrophobic/small amino acids, ξ hydrophilic amino acids, [+] positively charged amino acids, x any amino acid. (**C**) The amino acid sequence of the indicated proteins was aligned and the percent identity is shown.

Seven of the nine putative porins (Alr0834, Alr2231, All4499, Alr4550, Alr4741, All7614, Alr3608) contain an S-layer homology domain (SLH) which is also found in other cyanobacterial porins [19]. In Alr3608, the two predicted SLH-domains are located in the C-terminal region, whereas in other porins they are found in the N-terminal part of the protein (Fig. 1A). Moreover, seven of the putative porin proteins contain an OPR-B domain that is characteristic for carbohydrate-selective porins (Fig. 1A). This domain could not be identified in Alr3608 and Alr3917. In turn, a domain of unknown function (DUF) and a short region characteristic for a DNA translocase FtsK were identified in Alr3608 and Alr3917, respectively. However, a BLAST search with the Alr3917 sequence against cyanobacterial genomes in the NCBI-database did not yield a similarity to cyanobacterial FtsK sequences. Notably, *Anabaena* sp. FtsK is encoded by *all7666*.

The comparison of the amino acid sequence of the last β-strand in the seven porins with OPR-B fold shows a high degree of conservation, while Alr3608 and Alr3917 are somewhat distinct (Fig. 1B). It is proposed that the most C-terminal region of proteobacterial outer membrane proteins with β-barrel fold contains the signal for membrane insertion and Omp85 interaction [51-54]. In addition, the so called β-signal initiates the association with Omp85 (BamA; [55, 56]). The comparison of the identified motif to that of the 22 TonB-dependent transporters (TBDT) in the outer membrane uncovers a common motif in the last β-strand: hydrophobic/small amino acid (Φ) – x – Φ – x – hydroxylated/ hydrophobic/small amino acids (Φ/η) – x – aromatic amino acids (Ω) – x – Ω.. This motif might be important for the insertion of the proteins into the outer membrane, as it was reported that an aromatic amino acid at the last position of the sequence is important for proteobacterial outer membrane protein insertion [53]. In addition, the insertion of mitochondrial β-barrel proteins depends on a hydrophobic signal in the last strand as well [57]. Comparison of the amino acid sequence of the seven porin-like proteins to the amino acid sequence of the two most abundant *E. coli* porins, namely OmpC and OmpF [5], yielded a sequence identity between 18.8% to 24.6% (Fig. 1C). This indicates that the cyanobacterial proteins do not share a high sequence similarity to proteobacteriual porins.

In summary, the bioinformatics inspection revealed that seven out of the nine predicted porins in *Anabaena* sp. contain a domain architecture consistent with a porin-like function, although no secretion signal was predicted for Alr4741. The other two proteins previously assigned to the porin-like family (Alr3608, Alr3917) contain a domain architecture that is rather atypical for porins. As it is questionable whether these two proteins constitute porins, they were excluded from the subsequent analyses.

### Genotyping of Porin Mutants and Growth under Standard Conditions

**Fig. 2:**
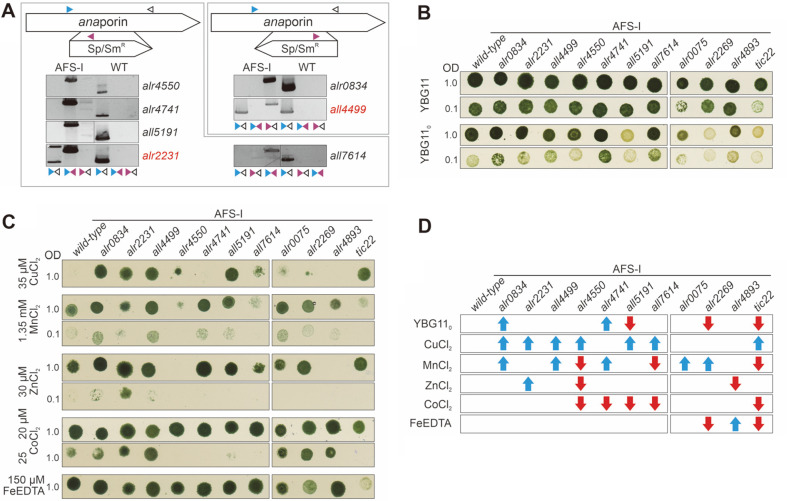
Metal sensitivity of porin mutants. (**A**) Genotype of the porin-insertion mutants. The orientation of the cassette (on top) and annealing position of oligonucleotides (colored arrowheads) are indicated. Panels show PCR products on gDNA of mutant strains (lane 1−3; AFS-I) or wild type (lane 4−6; WT) using the gene specific forward (blue) or reverse (black), or the plasmid specific oligonucleotide (magenta) as indicated. Mutants that are not segregated because a wild type gene fragment could be amplified are highlighted in red. (**B**, **C**) 5 μl of wild type and mutant strains (names on top) at OD_750_ = 1.0 (or 0.1 if indicated) were spotted on plates of YBG11 (**B**), YBG11_0_ (**B**) or YBG11 containing indicated concentrations of divalent metals (**C**). Representative images after 7 days of growth are shown, the test was conducted three times. (**D**) The increased (blue arrow) or decreased (red arrow) growth capacity of a mutant strains on YBG11_0_ or YBG11 with the indicated ingredient in comparison to wild type is presented as model.

In order to assess specific mutant phenotypes, single-recombinant mutants were created by integration of a plasmid bearing the CS.3 cassette into the porin genes. Five mutants had the plasmid insertion in forward direction, while the plasmid was inserted in the opposite direction into *alr0834* and *all4499* loci (Fig. 2A). Mutants of *all4499* and *alr2231* were not segregated, as wild-type copies of the genes were present after repeatedly diluting the cultures on medium containing antibiotics (Fig. 2A, first lane). This might indicate an essential function of the two gene products under the given conditions. For all other genes, segregated mutants were obtained.

The growth behavior of the porin mutants was compared to wild type. In addition, mutants exhibiting alterations in outer membrane integrity, bearing either an insertion in *Anabaena* sp. *omp85* genes (AFS-I-*alr0075*, AFS-I-*alr2269* and AFS-I-*alr4893*; AFS stands for *Anabaena* mutant created in Frankfurt by the Schleiff group)[58] or the *tic22* gene (AFS-I-*tic22*, *alr0114*, [45, 46, 59]) were used as additional controls. As reported before, AFS-I-*alr0075*, AFS-I-*alr2269* and AFS-I-*alr4893* could not be segregated since wild-type copies could be amplified even after exposing the strains to increased concentrations of antibiotics [58] (Fig. S1).

On standard YBG11 plates only AFS-I-*alr0075* and AFS-I-*tic22* grew to a lower density compared to wild type (Fig. 2B, first and second panel). This behavior was not described before [45, 46, 59] as the effect was only visible when the culture was sufficiently diluted (Fig. 2B, second panel). However, the strains with plasmid insertion in the porin-like genes did not show any phenotype under this condition (Fig. 2B).

### The Mutants of Three Porin-Like Genes Are Affected in Growth in Absence of Combined Nitrogen

On plates without a combined nitrogen source (YBG11_0_), a condition where *Anabaena* sp. fixes atmospheric nitrogen in heterocysts, AFS-I-*alr2269* and AFS-I-*tic22*, but not AFS-I-*alr0075* and AFS-I-*alr4893* were impaired in growth (Fig. 2B, third panel) as previously reported [46]. With respect to the with respect to the porin mutants, AFS-I-*all5191* grew to a lower density than wild type on YBG11_0_ plates (Fig. 2B, third panel). In addition, AFS-I-*alr4741* and AFS-I-*alr0834* grew to slightly higher densities under diazotrophic conditions compared to wild type.

**Fig. 3:**
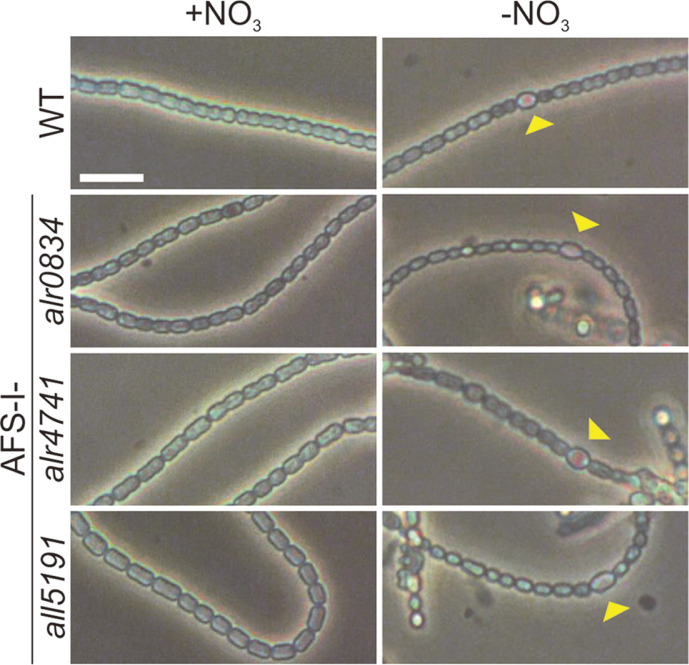
Filaments of wild type (WT) and the three mutant strains AFS-I-*alr0834*, AFS-I-*alr4741* and AFS-I*all5191*. Strains were grown in the presence or absence of nitrate (YBG11 and YBG11_0_, respectively). Heterocysts observed in absence of nitrate are marked with a yellow arrowhead. The reference bar indicates 5 μm.

Consequently, filaments of wild type, AFS-I-*alr0834*, AFS-I-*alr4741* and AFS-I-*all5191* grown in YBG11 or YBG11_0_ were microscopically inspected. In standard YBG11 medium the filaments of all strains appeared morphologically comparable to wild-type cells (Fig. 3, +NO_3_). Heterocysts were not detected in the mutant and wild-type strains grown in the presence of nitrate. After a seven-day cultivation in YBG11_0_ medium heterocysts were visible in wild type, AFS-I-*alr0834*, AFS-I-*alr4741* and AFS-I-*all5191* (Fig. 3, -NO_3_). Thus the defective growth of AFS-I-*all5191* is not due to an inability of this strain to differentiate heterocysts. Though, since in general the cells of AFS-I-*all5191* looked comparatively pale in the absence of nitrate (Fig. 2B), we assume that nitrogen fixation might not work properly in this strain.

The heterocyst pattern was determined by counting the vegetative cells between two heterocysts in the three strains and significance of the difference to wild type was assessed with Student’s t-test. In AFS-I-*alr0834* and AFS-I-*alr4741* on average 16.2 ± 4.6 and 16.7 ± 4.7 vegetative cells were found between two heterocysts respectively (>1500 cells were counted for each strain). Hence there was no significant difference compared to wild type, where on average 15.4 ± 6.4 vegetative cells existed between heterocysts (n > 600 cells). In AFS-I-*all5191* however on average 11.8 ± 4.6 vegetative cells were found between two heterocysts (n > 1200 cells) after 7 days of growth without nitrate, which significantly differs from the wild type (Student’s t-test *p* = 4.3 × 10^-6^). This suggests a necessity of AFS-I-*all5191* to increase the relative number of heterocysts per filament to compensate for either an inefficient nitrogen fixation or an increased demand of the strain.

### Most of the Genes Coding for Porin-like Proteins are Upregulated by Metal Starvation

The regulation of porin-mediated solute diffusion in bacteria is governed by distinct parameters. For instance, the extracellular nutrient supply status and the osmolarity might trigger transcriptional responses [60]. *Anabaena* sp. performs photosynthesis and is therefore not obligatorily dependent on sugar uptake. Thus, the potential changes in gene expression of porins in response to altered metal concentrations in the growth medium were examined. For this, *Anabaena* sp. wild type was grown in YBG11 with modified metal concentrations by omitting either manganese (Mn), iron (Fe), zinc (Zn) or copper (Cu) from the medium. These metals were used since *Anabaena* sp. accumulates (5.0 ± 0.3) × 10^14^ atoms Mn in cells equivalent to 1 ml culture at OD_750_ = 1, (0.5 ± 0.05) × 10^14^ atoms Cu/1 ml (OD_750_ = 1) and (0.5 ± 0.03) × 10^14^ atoms Zn/1 ml (OD_750_ = 1) as determined by ICP-MS. By that, the cellular concentration of these metals is in the similar range to that of iron ((3.4 ± 0.2) × 10^14^ atoms Fe/1 ml (OD_750_ = 1)), which is considered to be a limiting factor for cyanobacterial growth and is especially important for N_2_-fixing cyanobacteria [61, 62].

Thus, RNA was isolated from wild-type cells grown for 21 days in the indicated media (Table 1) and qRT-PCR was performed on cDNA. The high transcript abundance of *alr4550* in standard YBG11 medium corresponds to proteomic analyses in which Alr4550 and All4499 were identified as the highest abundant porins in the OM of *Anabaena* sp. [30].

The mRNA abundance of *all7614* was not affected after 21 days of culturing wild type in media lacking either manganese, iron, copper or zinc (Table 1). Manganese deprivation resulted in an increase of the transcripts of *alr0834*, *alr2231*, *alr4741* and *all5191* when compared to YBG11 (Table 1). The transcript of *alr0834* was further increased under iron limitation, whereas the transcript of *all5191* was increased under iron and zinc limitation. Moreover, iron deprivation yielded in an elevated *all4499* transcript level when compared to cultivation in YBG11.

Notably *alr4550* demonstrated an exceptional behavior. On the one hand, it is highly expressed in YBG11 when normalized to *rnpB*. On the other hand, among all tested genes, only *alr4550* transcripts decreased in medium lacking Fe, Cu or Zn (Table 1). Only Mn deprivation did not significantly alter *alr4550* transcript levels (Table 1).

In conclusion, the expression of the genes coding for the seven porin-like proteins does not show a common regulation after 21 days in media without individual trace metals. This might indicate distinct functions of the analyzed proteins. Altogether, manganese and iron depletion resulted in increased abundance of four and three transcripts, respectively. Zn depletion led to enhanced mRNA levels of only *all5191*.

### The Mutants of Porin-like Genes Show a Differential Sensitivity to Divalent Metal Stress

The sensitivity against elevated metal concentrations was tested with the porin mutants, as it is expected that strains with defected transport capacities of certain substrates display hyper-resistance towards increased concentrations of these substrates. Interestingly, virtually all mutant strains except AFS-I-*alr4741* exhibited a resistance towards elevated copper concentrations (35 μM) compared to the wild type. AFS-I-*alr4550* and AFS-I-*all7614* grew to a slightly lower density under those conditions compared to the other mutant strains (Fig. 2C, first panel). Also, the growth of the two *omp85* mutants, AFS-I-*alr2269* and AFS-I-*alr4893*, was inhibited under elevated copper concentrations, while AFS-I-*alr0075* grew to a lower density (Fig. 2C, first panel). Thus, the *omp85* mutants exhibited a similar sensitivity against the selected copper concentration compared to wild type, while AFS-I-*tic22* showed an enhanced resistance. It can be concluded that copper entry into the porin mutants is largely limited compared to wild type. Notably, none of the porin transcripts was increased after copper depletion, leading to the assumption that this effect might not be specifically related to a single porin (Table 1).

On plates with an excess of manganese (1.35 mM MnCl_2_; Fig. 2C, second & third panel) AFS-I-*alr4550* and AFS-I-*all7614* grew to lower densities when compared to wild type, while AFS-I-*alr0834*, AFS-I-*all4499* and AFS-I-*alr4714* appeared to be more resistant as they grew to higher densities. AFS-I-*alr0075* and AFS-I-*alr2269* were more resistant than wild type, while AFS-I-*tic22* showed a higher sensitivity (Fig. 2C, second & third panel). Interestingly AFS-I-*alr2231* grew better than wild type when the zinc concentration was enhanced (Fig. 2C, fifth panel). With respect to the control strains, only AFS-I-*alr4893* was hyper-sensitive towards this zinc concentration (Fig. 2C, fourth panel).

In addition to the mentioned divalent metals, the growth in the presence of enhanced cobalt concentrations was determined as well. The ICP-MS measurements showed that cobalt was about ten-fold less abundant in cells then the other metals ((1.79 ± 0.04) × 10^13^ atoms Co/1 ml (OD_750_ = 1)). AFS-I-*alr4550*, AFS-I-*alr4741*, AFS-I-*all5191* and AFS-I-*all7614* were hypersensitive towards 25 μM CoCl_2_ (Fig. 2C, seventh panel), but not towards 20 μM CoCl_2_ (Fig. 2C, sixth panel). Notably AFS-I-*tic22* was already reduced in growth when 20 μM CoCl_2_ was present (Fig. 2C, sixth panel). On plates with an excess iron (150 μM FeCl_2_-EDTA) no clear phenotype could be observed for any of the tested porin mutants, while AFS-I-*alr2269* and AFS-I-*tic22* were more sensitive to the elevated iron levels than wild type (Fig. 2C, eighth panel).

Taken together most porin mutants were more resistant to enhanced copper concentrations when compared to wild type, while their behavior in response to enhanced iron levels was comparable to wild type (Fig. 2D). In *Anabaena* sp. ferric iron chelates typically get transported by TBDTs, however the expression of the siderophore transport system gets triggered mainly under starvation conditions [63]. It is hypothesized that soluble iron diffuses through porins under replete conditions, as shown for the unicellular cyanobacterium *Synechocystis* sp.[27]. Remarkably, only AFS-I-*alr2231* was more resistant towards high zinc concentrations, which might lead to the assumption that Alr2231 facilitates zinc transport. To support this conclusion, the expression of other porins in this mutant strain was analyzed. Interestingly, the transcript abundance of all other porin-coding genes was increased in this mutant (Table 2). This strengthens the hypothesis that zinc resistance is mediated by *alr2231*-insertion and not by the diminished transcription of another putative porin gene.

In addition, in the presence of enhanced concentrations of manganese, the mutants of *alr0834*, *all4499* and *alr4741* were more resistant than wild type. Interestingly, *all4499* was comparatively high expressed under standard conditions and the transcript abundance did not change after manganese deprivation. However, the expression of *alr0834* and *alr4741* was upregulated in the absence of manganese (Table 1). Analyzing the transcript abundance of the other porins in the respective mutants revealed a downregulation of *alr4550* and *all7614* expression in AFS-I-*alr0834* (Table 2). The mutants of *alr4550* and *all7614* were the only strains showing a hypersensitivity towards an elevated manganese concentration. Therefore, this downregulation does not explain the manganese resistance of AFS-I-*alr0834*. In AFS-I-*all4499* and AFS-I-*alr4741* no other gene coding for a porin-like protein was found to be downregulated (Table 2). Thus, the resistance of AFS-I-*all4499* and AFS-I-*alr4741* against elevated manganese can be attributed to the mutated gene.

### The Mutant of *alr4550* Shows a Reduced Integrity of the Outer Membrane

**Fig. 4:**
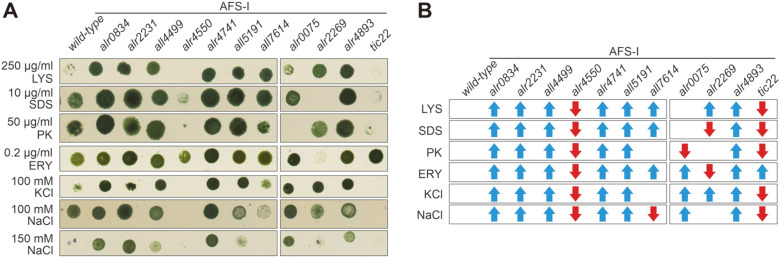
Outer membrane integrity of porin mutants. (**A**) 5 μl of wild type, the porin, the *omp85* and the *tic22* insertion mutants (indicated on top) at OD_750_ = 1.0 were spotted onto media composed of YBG11 supplemented with indicated divalent metals. Images were taken after 7 days of growth. Representative results (*n* = 3) are shown. (**B**) The increased (blue arrow) or decreased (red arrow) growth capacity of a mutant strain on YBG11 with the indicated ingredient in comparison to wild type is indicated. LYS = lysozyme, SDS = sodium dodecyl sulfate, PK = proteinase K, ERY = erythromycin.

Next, the integrity of the outer envelope was analyzed in the porin mutants. To this end, lysozyme that catalyzes peptidoglycan hydrolysis was added to the medium (Fig. 4A, first panel). AFS-I-*alr4550*, and AFS-I-*tic22* were hampered in growth in presence of 250 μg ml^-1^ lysozyme when compared to wild type. For AFS-I-*tic22*, this is in line with earlier findings [46]. In turn, AFS-I-*alr0075* grew similar to wild type, while all other porin mutants showed an improved growth compared to wild type. This was observed as well for the *omp85* mutants AFS-I-*alr2269* and AFS-I-*alr4893*. On plates containing 10 μg ml^-1^ SDS, the porin mutants followed a comparative trend as in presence of lysozyme; the porin mutants grew somewhat better than wild type, which in turn did grow better as in presence of lysozyme (Fig. 4A, second panel). Here AFS-I-*alr4550* did grow, but only to a low density. The mutants AFS-I-*alr2269* and AFS-I-*tic22* were inhibited in growth in the presence of SDS, while AFS-I-*alr4893* was less affected than wild type. In the presence of 50 μg ml^-1^ proteinase K the porin mutants behaved again similar as in the presence of lysozyme, while only AFS-I-*all7614* behaved similar to wild type (Fig. 4A, third panel). AFS-I-*alr0075* and AFS-I-*tic22* were inhibited in growth in presence of proteinase K. Instead, AFS-I-*alr2269* growth was comparable to wild type and AFS-I-*alr4893* grew better than wild type. In presence of the antibiotic erythromycin again all porin mutants except AFS-I-*alr4550* showed an enhanced growth compared to wild type (Fig. 4A, fourth panel), with the exception of AFS-I-*alr2269* which exhibited growth inhibition.

Next, the cells were grown under salt stress, which is known to alter the abundance of porins [64]. In the presence of 100 mM KCl a similar growth of the porin mutants as in the presence of proteinase K was observed, while the growth of the *omp85* mutants was enhanced. Growth of AFS-I-*tic22* on the other hand was severely affected (Fig. 4A, fifth panel). The addition of 100 mM NaCl reduced the growth of AFS-I-*alr4550*, AFS-I-*all7614* and AFS-I-*tic22* when compared to wild type (Fig. 4A, sixth panel). When 150 mM NaCl was added, wild type, AFS-I-*alr4550*, AFS-I-*all7614*, AFS-I-*alr2269* and AFS-I-*tic22* were defective in growth, while AFS-I-*alr0834*, AFS-I-*alr2231*, AFS-I-*all4499*, AFS-I-*alr4714*, AFS-I-*all5191*, AFS-I-*alr0075* and AFS-I-*alr4893* were able to grow on this medium (Fig. 4A, seventh panel).

Our results demonstrate that the mutants of five porin-like genes (*alr0834*, *alr2231*, *all4499*, *alr4741* and *all5191*) were more resistant towards the selected compounds, which is indicative for alterations in the outer membrane integrity. AFS-I-*tic22* for instance, a previously characterized mutant with alterations in OM biogenesis, exhibited severe growth defects (Fig. 4). In contrast, mutation of the highest expressed porin-like gene *alr4550* causes a phenotype that is consistent with an impaired outer membrane integrity in general (Figs. 2 and 4). AFS-I-*all7614* showed an intermediate phenotype. On the one hand, the strain was sensitive to divalent metals and sodium chloride (Figs. 2 and 4), on the other hand the mutant presented a higher resistance to SDS and lysozyme.

### Omp85 Proteins are Distinct in Their Function In Porin Biogenesis

Altogether these results can be seen as first hint towards a putative functional relation between the Omp85 protein Alr4893 and most of the porins, as the mutant phenotypes were consistent under many conditions (Figs. 2 and 4). In order to assess the influence of the *omp85* gene mutations on the porins, the expression of the putative porin-coding genes was analyzed in the three *omp85* mutants (Table 3). In AFS-I-*alr0075* a reduction of the transcripts of *all4499* and *all5191* was observed. In AFS-I-*alr2269* a reduction in transcript abundance of *alr0834* and *all5191* was detected, while the abundance of *all7614* was enhanced (Table 3). In turn, the transcript abundance of *all7614* was strongly reduced in AFS-I-*alr4893*, while the transcript levels of *alr4550* and *alr4741* were increased. The mRNA levels of *alr2231* were not affected by the mutation of *omp85* genes.

**Fig. 5:**
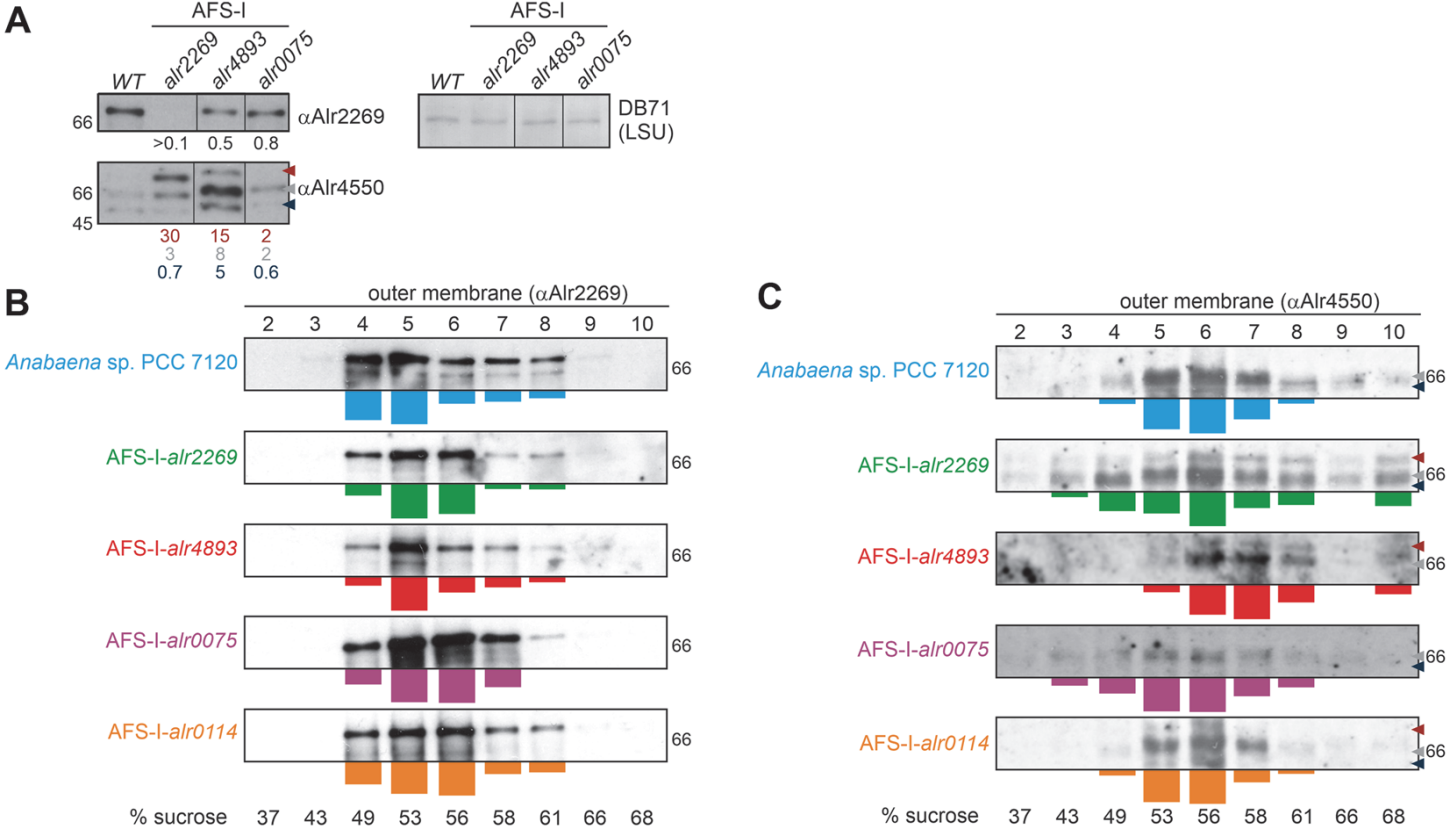
Relation between Omp85 function and porin biogenesis. (**A**) A representative result (*n* = 3) for the outer membrane protein content in wild type (first lane) and the three indicated *omp85* mutants (lane 2-4) is shown. Total protein extract was probed with specific antibodies against Alr2269 or Alr4550 as indicated. DB71 staining of the large subunit of Rubisco (LSU) was used as loading control. The average of the ratio of the protein density in mutants and wild type is shown as analyzed with ImageJ. The standard deviation is smaller than 25%. All lanes come from the same gel and have been developed simultaneously. (**B**, **C**) Outer membrane vesicles were isolated from the indicated strains and subjected to rate zonal ultracentrifugation. Fractions 2-10 of the sucrose gradient (25% to 70% (v/w)) were probed with an antibody against Alr2269 (**B**) or Alr4550 (**C**). Note: in B the result for AFS-I-*alr2269* was longer exposed than other blots to visualize the bands. In (**B**) and (**C**) one of two repetitions is shown. The densitometric protein distribution of both experiments normalized to the highest intensity is shown on the bottom of each panel as bar diagram. In (**A**) and (**C**) the blue arrowhead marks the presumed degradation product of Alr4550, the grey arrowhead marks the full length protein and the red arrowhead points at a fragment that likely represents a non- native state of the protein. The migration of the 66 kDa molecular weight standard is indicated.

To determine the importance of the Omp85 protein function for porin-insertion into the outer membrane, the protein abundance of a porin was examined in wild type and the *omp85* mutants. As Alr4550 was found to be the most abundant protein component of the *Anabaena* sp. outer membrane [30] the abundance of this protein was tested. Total protein was extracted from wild type, AFS-I-*alr0075*, AFS-I-*alr2269* and AFS-I-*alr4893*. The presence of outer membranes in the total protein fractions was confirmed by detection of Alr2269 using a specific antibody. The protein was detected in the cell lysate of all strains except AFS-I-*alr2269* (Fig. 5A, panel 1). Thus, Alr2269 could not be detected in AFS-I-*alr2269* lysate although the strain is not segregated (Fig. S1). The relatively small amount of outer membrane proteins in total cell lysate and the reduced protein abundance in the mutant are possible explanations for this. The loading of lysate was comparable between all four strains as controlled by DB71 staining (Fig. 5A, panel 3, LSU). Probing for Alr4550 in wild type resulted in two apparent signals (Fig. 5A, panel 2). The upper band corresponds to the full length protein, whereas the lower molecular weight signal most likely represents a degradation product. Both fragments have been detected in wild type in previous experiments [45]. The size of the lowest fragment is consistent with the molecular weight of Alr4550 lacking the periplasmic S-layer homology domain.

Alr4550 was more abundant in the *omp85* mutants AFS-I-*alr2269* and AFS-I-*alr4893* compared to the wild type. Especially in AFS-I-*alr4893* the protein amount was comparatively increased. Moreover, the protein band of higher molecular weight than the mature protein was more abundant in the mutants when compared to wild type (Fig. 5A, panel 2). This apparent fragment likely represents an SDS-resistant unfolded or aggregated intermediate, which likewise has been reported for other outer membrane β-barrel proteins [65, 66]. In AFS-I-*alr2269* the high molecular weight fragment was the dominating species, whereas in AFS-I-*alr4893* the form migrating as intermediate predominated. Hence, our results might suggest that the plasmid-insertion in *alr2269* or *alr4893* leads to an increased protein production of Alr4550 as well as an increased detection of misfolded or degraded proteins. The increase in Alr4550 protein amount was found to be reflected by an enhanced mRNA abundance of *alr4550* in AFS-I-*alr4893*, but not AFS-I-*alr2269* (Table 3).

Alterations in the outer membrane integrity and biogenesis as well as an alteration of the protein to lipid ratio can result in an aberrant membrane density which can be examined by rate zonal centrifugation [67-69]. The effects of the *omp85* mutations on membrane protein integrity were examined by analyzing the outer membrane protein density in the single insertion mutants. The sedimentation behavior of outer membranes was analyzed by rate zonal centrifugation. Gradient fractions were collected and subjected to SDS-PAGE. To detect non-porin type OM-proteins the antibody against Alr2269 was utilized (Fig. 5B). As AFS-I-*alr2269* is not segregated a signal was obtained in this sample as well.

Isolated outer membranes from *Anabaena* sp. that contained Alr2269 sedimented in the range of 49% to 61%sucrose. The largest quantity of Alr2269 was detected between 49% and 53% (Fig. 5B, top panel). The same distribution of Alr2269 was detected when outer membrane preparations of the *omp85* mutants or AFS-I-*tic22* were analyzed. However, in AFS-I-*alr0075*, AFS-I-*alr2269* and AFS-I-*tic22* the majority of the protein migrated between 53% and 56% sucrose (Fig. 5B).

In wild type outer membrane fractions Alr4550 was more broadly dispersed than Alr2269. Similar to Alr2269, the largest quantity of Alr4550 was found between 49% to 61% sucrose as well. The peak was shifted to the fractions between 53% and 56% sucrose (Fig. 5C, top). Interestingly, the distribution of Alr4550 in the membrane fractions isolated form AFS-I-*alr0075* (Fig. 5C, fourth panel) and AFS-I-*tic22* (Fig. 5C, bottom) was comparable to that of wild type. Hence, Alr4550 synthesis and integrity seems not affected in AFS-I-*alr0075*. This is also underlined by the fact that the Alr4550-fragment which putatively represents the aggregated form of the protein was not detectable in AFS-I-*alr0075* (Fig. 5A and 5C, fourth panel). In turn, in AFS-I-*tic22* the aggregated form was detectable (Fig. 5C, bottom, red arrowhead).

The sedimentation behavior of Alr4550 in samples from AFS-I-*alr2269* and AFS-I-*alr4893* showed clear distinctions compared to wild type. While the general distribution profile of the membrane fractions containing Alr4550 from AFS-I-*alr2269* was more disperse than in wild type (ranging from 43% to 61%), the peak fraction was found at a comparable density (Fig. 5C, second panel). However, an enrichment of the porin in fractions of higher density that possibly represents aggregated protein was observed (Fig. 5C, second panel, fraction 10). The migration of the membrane fractions isolated from AFS-I-*alr4893* that contained Alr4550 was shifted to higher density compared to wild type (56%−61% sucrose; Fig. 5C, third panel). Just like observed in AFS-I-*alr2269* samples, a significant portion of Alr4550 was found in the last gradient fractions. In both strains the high molecular-weight fragment that likely represents the aggregated form was identified, whereas in wild type samples it was not detected in this experiment. This is consistent with earlier reports documenting the aggregation of unfolded, not inserted proteins at the outer membrane [70].

Taken together these results indicate that outer membrane biogenesis or integrity is affected in AFS-I-*alr0075*, AFS-I-*alr2269* and AFS-I-*tic22*, as judged from the sucrose gradient centrifugation analysis and detection of Alr2269 and Alr4550, whereas AFS-I-*alr0075* samples only exhibited an aberrant distribution of Alr2269, but not Alr4550. Factors that could cause the increased density of the vesicles could be for example an increased protein to lipid ratio, an altered (enhanced) co-migration of peptidoglycan with the proteins or variations in LPS production [69, 71, 72].

## Discussion

Porins have an important function in the regulation of solute uptake and further contribute to the maintenance of envelope integrity [8]. The degree of specificity of certain porin classes is highly diverse [6]. In proteobacteria a distinction between *e.g.* OmpA-type porins (structural function) and OmpF-type porins (transport function) can be made [8]. In the genome of the filamentous cyanobacterium *Anabaena* sp. a typical OmpA-like porin, namely Alr4550, was identified. Expression analysis revealed that among all the porins in *Anabaena* sp. the *alr4550*-transcript was high abundant under standard conditions (Table 1). Remarkably AFS-I-*alr4550* was hypersensitive compared to wild type under virtually all tested conditions (except elevated copper levels). Growth of AFS-I-*alr4550* in the absence of fixed nitrogen was not inhibited, hence Alr4550 dysfunction seems not to affect heterocyst development (Figs. 2 and 3). Thus, Alr4550 might have a prominent structural function in *Anabaena* sp. that is distinct from the function of the other porin-like proteins, considering the exceptionality of the mutant phenotype. This could be for instance mediated by the interaction of Alr4550 with cell wall (components) and thereby connecting the OM to the peptidoglycan, as described for OmpA in *E. coli* [49, 73].

Most of the porin mutants were increasingly resistant towards elevated potassium chloride and sodium chloride concentrations compared to the wild type. The same was observed when the macrolide erythromycin was added. In contrast to other antibiotics, macrolide antibiotics are generally not thought to penetrate through porins, therefore the enhanced resistance might point towards altered membrane properties (Fig. 3) [18, 74]. Interestingly AFS-I-*all5191* was the only strain that did barely grow in the absence of a combined nitrogen source (Fig. 2). Apparently, this strain was able to differentiate heterocysts as judged from microscopic analyses, showing that the protein might not play a role in heterocyst development. We rather suggest that the absence of All5191 generates a condition that complicates nitrogen fixation. Consequently, the percentage of heterocysts increases is AFS-I-*all5191* filaments. AFS-I-*alr0834* and AFS-I-*alr4741* grew better on YBG11_0_ compared to wild type (Fig. 2). An alteration in the heterocyst pattern of the two strains was not observed, thus the reason for the enhanced growth of the mutants remains to be elucidated. Noteworthy the transcript of *all5191* was increasingly abundant in AFS-I-*alr0834* and AFS-I-*alr4741*. Since AFS-I-*all5191* was defected in growth in absence of a combined nitrogen source, an increased production of the protein might on the other hand have beneficial effects on the growth capacity in YBG11_0_, as observed in AFS-I-*alr0834* and AFS-I-*alr4741*.

With respect to the uptake of divalent ions, a relation to iron uptake could not be established in this study. Ferric iron ions are prevalently complexed to organic ligands like siderophores [75]. However, the transport of siderophores in *Anabaena* is rather dependent on functional TBDTs instead of porins [63, 76]. It was shown that inorganic iron is highly bioavailable to cyanobacteria [77, 78]. Moreover, homologues of the iron-transporting porin Slr1908 in *Synechocystis* sp. are found in many freshwater and marine cyanobacteria species [27]. This generally suggests an important role of porin-mediated iron transport among cyanobacteria, which needs to be further examined in *Anabaena* sp.

Also for cobalt uptake, no relation to a porin in *Anabaena* sp. could be made, as mutation of porins did not lead to a resistance against elevated concentrations (Fig. 2). Cobalt also might be taken up in *Anabaena* sp. in form of cobalamin. This uptake is dependent on the BtuB proteins of the TBDT-family, which are predicted to exist in *Anabaena* sp. as well [79]. Similarly, two TBDTs but none of the porin-like genes investigated here have been found to be regulated by the zinc starvation sensor Zur in *Anabaena* sp. [80]. This is consistent with the earlier identification of the zinc transporter ZnuD in proteobacteria, which belongs to the TonB-dependent transporter (TBDT) family [81, 82]. However, AFS-I-*alr2231* shows a high resistance to elevated levels of zinc (Fig. 2), which might link the Alr2231 function to the uptake of zinc when the trace metal is present in sufficient amounts. Such function would be consistent with the identification of a cyanobacterial porin as zinc binding protein [83]. An adequate zinc supply is important for organisms as for example the carbonic anhydrase depends on zinc. This enzyme is involved in conversion of CO_2_ to bicarbonate, which is important for the regulation of the pyruvate conversion to oxaloacetate and thus central for the cyanobacterial metabolism.

The uptake of other divalent metals like manganese and copper is discussed to depend on porin-like proteins in Gram-negative bacteria [84–88]. The importance of manganese is associated with the regulation of the pyruvate pool as phosphoenolpyruvate carboxykinase and pyruvate carboxylase are Mn-dependent metalloenzymes. Moreover, the oxygen evolving complex in photosystem II bears manganese as cofactor [89]. In turn, the manganese content is tightly regulated and 50 fold excess leads to growth inhibition [90]. Three porin mutant strains were resistant against enhanced manganese concentrations, namely AFS-I-*alr0834*, AFS-I-*alr4741* and AFS-I-*all4499* (Fig. 2). Interestingly, *alr0834* and *alr4741* are upregulated in the absence of manganese, while *all4499* belongs to the three most abundant transcripts among the porins (Table 1). No significant downregulation of the other two genes was observed in the three mutants (Table 2), this suggests that the three porins might act in parallel in manganese uptake. The interplay between these three porins remains to be further explored.

Copper is a globally important co-factor, *e.g.* for respiration and photosynthesis. Consistent with reports for other bacteria [84, 88] most of the mutant strains showed an increased survival on medium with high copper concentrations (Fig. 2). Thus, many porins might be involved in copper uptake under replete conditions. In contrast, for *Anabaena* sp. the TBDT IacT was identified which might be involved in copper uptake under highly limiting conditions [91].

Interestingly, only the *tic22*-mutant, but not the three *omp85* mutants exhibits an elevated resistance towards high copper concentrations (Fig. 2). Omp85 proteins are involved in membrane insertion of OM-proteins [92,93] and Tic22 acts as periplasmic shuttle [45]. The phenotype of AFS-I-*alr4893* showed the highest accordance to the phenotypes of most porin mutants (except to that of AFS-I-*alr4550*, Fig. 4), which could suggest a direct function of Alr4893 in porin insertion.

Moreover, in AFS-I-*alr2269* and AFS-I-*alr4893* the accumulation of the denatured form and a high abundance of aggregated Alr4550 were observed. Based on these results two interpretations are possible, which need to be explored in future. One could suggest that both Alr2269 and Alr4893 are involved in membrane insertion of porins, which would explain the rather moderate correlation between the phenotypes of porin mutants and *omp85* mutants. Alternatively, the data would also be consistent with a functional diversification of the two Omp85 proteins. By this means Alr2269 could be specialized on the insertion of larger OM-proteins such as members of the Omp85 family, LptD or the TBDTs. On the other hand, Alr4893 could be specifically inserting porins into the OM. This could explain the observed diversity in the C-terminal amino acid composition between TBDTs and porins (Fig. 1) and the high correlation of the phenotypes of the porin mutants with AFS-I-*alr4893*, but not AFS-I-*alr2269*. The occurrence of the aggregated porin form in AFS-I-*alr2269* would be the consequence of a reduced insertion of Alr4893. Both explanations suggest that Alr0075 only plays a minor role in porin insertion. Thus, Tic22 might globally act as periplasmic transfer protein including porins as substrate.

Our results imply diverse functions of single porin-like proteins, reflected by the specific phenotypes of the mutants. Most prominently AFS-I-*alr4550* exhibited a unique phenotype by sowing hypersensitivity towards metals, salt and harmful compounds. Also the strong transcription of *alr4550* suggests a crucial function of the protein, which apparently involves the maintenance of envelope integrity. First associations between single porin candidates and manganese, zinc or copper transport as well as diazotrophic growth capacity could be drawn, thus these connections need to be further examined. The density gradient centrifugation experiments showed that the three *omp85* mutants in *Anabaena* sp. are affected in the migration of Alr2269 and Alr4550, but to different extents. The presence of aggregated intermediates of Alr4550 in the two *omp85* mutants AFS-I-*alr2269* and AFS-I-*alr4893*, but not in AFS-I-*alr0075*, suggests a functional specification on certain substrates for membrane insertion.

## Supplemental Materials



Supplementary data for this paper are available on-line only at http://jmb.or.kr.

## Figures and Tables

**Table 1:** qRT-PCR analysis of expression of porin genes.

	*Anabaena* sp. PCC 7120					

	YBG11	YBG11_-Mn_	YBG11_-Fe_	YBG11_-Cu_	YBG11_-Zn_

	-ΔCt	-ΔΔCt	-ΔΔCt	-ΔΔCt	-ΔΔCt
*alr0834*	-10.8 ± 0.3	**1.7 ± 0.1**	**1.6 ± 0.1**	0.1 ± 0.2	0.8 ± 0.1
*alr2231*	-12.3 ± 0.3	**1.9 ± 0.1**	0.0 ± 0.1	0.5 ± 0.3	0.9 ± 0.1
*all4499*	-9.4 ± 0.2	0.5 ± 0.1	**1.4 ± 0.2**	-0.6 ± 0.2	0.2 ± 0.2
*alr4550*	-6.4 ± 0.1	-0.5 ± 0.1	***-3.5 ± 0.2***	***-3.3 ± 0.2***	***-2.8 ± 0.2***
*alr4714*	-11.5 ± 0.4	**1.3 ± 0.2**	0.2 ± 0.2	-0.3 ± 0.2	0.6 ± 0.2
*all5191*	-12.1 ± 0.5	**1.8 ± 0.1**	**1.1 ± 0.1**	0.0 ± 0.2	**1.7 ± 0.1**
*all7614*	-8.3 ± 0.2	0.3 ± 0.1	-0.5 ± 0.1	-0.8 ± 0.2	-0.3 ± 0.1

The -ΔCt value based on the housekeeping gene *rnpB* is given for YBG11 and the -ΔΔCt based on the YBG11 values is shown for the treatments, the standard deviation is indicated. Values in bold represent changes with *p* < 0.05 (Student’s *t*-test) and an absolute fold-change higher or lower (italics) than one.

**Table 2:** qRT-PCR analysis of porin genes in individual porin mutants.

	AFS-I-			

	*alr0834*	*alr2231*	*all4499*	*alr4741*

	-ΔΔCt			
*alr0834*	n.d.	**1.8 ± 0.4**	**4.4 ± 0.5**	0.9 ± 0.4
*alr2231*	0.7 ± 0.3	n.d.	0.9 ± 0.5	**2.8 ± 0.5**
*all4499*	-0.2 ± 0.1	**3.3 ± 0.4**	n.d.	0.2 ± 0.2
*alr4550*	**-1.5 ± 0.2**	**2.0 ± 0.4**	0.7 ± 0.3	-0.3 ± 0.1
*alr4741*	-0.1 ± 0.2	**2.5 ± 0.3**	0.0 ± 0.5	n.d.
*all5191*	**1.0 ± 0.1**	**2.2 ± 0.3**	0.7 ± 0.4	**1.3** **± 0.3**
*all7614*	**-2.5 ± 0.1**	**1.6 ± 0.4**	0.5 ± 0.2	0.1 ± 0.2

The -ΔΔCt based on the *rnpB* expression and BG11 values are shown for the mutants. Values in bold are changes with *p* < 0.05 (Student’s t-test) compared to wild type.

**Table 3:** qRT-PCR analysis of expression of porin genes in *omp85* mutants.

	AFS-I-		

	*alr0075*	*alr2269*	*alr4893*

	-ΔΔC		
*alr0834*	-1.2 ± 0.6	**-2.2 ± 0.1**	-0.2 ± 0.1
*alr2231*	-0.7 ± 0.1	-2.5 ± 2.1	0.2 ± 0.1
*all4499*	**-2.1 ± 0.6**	0.0 ± 0.1	0.3 ± 0.1
*alr4550*	-1.8 ± 1.2	0.7 ± 0.3	**2.0 ± 0.3**
*alr4741*	0.2 ± 0.1	0.5 ± 0.3	**1.4 ± 0.3**
*all5191*	**-2.3 ± 0.7**	**-2.3 ± 0.1**	-0.8 ± 0.5
*all7614*	-1.5 ± 0.9	**1.5 ± 0.1**	**-13 ± 11**

The -ΔΔC based on *rnpB* expression and BG11 values is shown for the mutants. Values in bold are changes with p < 0.05 (Student’s t-test) compared to wild type.
