# Supplementary material for: Comparative Phenotypic Analysis of Anabaena sp. PCC 7120 Mutants of Porinlike Genes
Source: J Microbiol Biotechnol. 2021 Apr 6;31(5):645–58. doi: 10.4014/jmb.2103.03009 (PMC9705863; doi:10.4014/jmb.2103.03009)
Supplement: Supplementary file 1 [file jmb-31-5-645-supple.pdf]

https://doi.org/10.4014/jmb.2103.03009

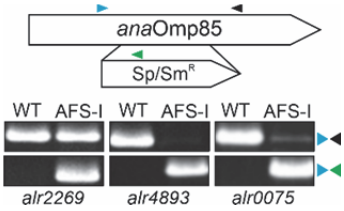

Figure S1. Segregation of *omp85* mutants. On top the orientation of the cassette is shown indicating the position of the primers used. The PCR product using gDNA isolated from the wild type (first lane, WT) or the indicated mutant strain (second lanes; AFS-I) using oligonucleotides flanking the recombination site (blue / black, upper panel) or using the gene specific forward and the cassette specific primer (blue, green) is shown. The strains are not segregated as judged from the occurrence of the wild-type gene with *alr2269* showing the lowest degree of segregation.

Table S1. Oligonucleotides used in this manuscript

purpose	name	sequence
mutagenesis	alr0834_FW	ATTTATAGATCTCAGCGATGTCTTATCAGGCG
	alr0834_RV	ATTTATAGATCTGGTCATTTGCTGTACCACTC
	alr2231_FW	AATTAAAGATCTGCATTGGCTAGTGTATTTGGC
	alr2231_RV	AATTAAAGATCTCGATGGGACAACGTAGCCAGCAG
	all4499_FW	AGATCTATCCCAACGGTACATACCGTGG
	all4499_RV	AGATCTGGATAGCATCGCTGAAGTTGAATGC
	alr4550_FW	AGATCTGGTTGAGCGCTACGGTTGTATTGC
	alr4550_RV	AGATCTCTGCTGCATAATGGCTATGTCG
	alr4741_FW	AGATCTGTGATTAGTGAAGATGCTC
	alr4741_RV	AGATCTGATATTAAGCCGATACG
	all5191_FW	AGATCTGGCACTACAAGCATTAAC
	all5191_RV	AGATCTCCAAAGCGTACAAAGTGACG
	all7614_FW	AGATCTGTTACATCAGTTTCC
	all7614_RV	AGATCTGAGAGTTGCCAACAGGGAAG
Screening of mutants	CS3_Rv	CTGATGCCGCATAGTTAAGCC
	alr0834_FW2	CTGCGGAACTAGCAAC
	alr0834_RV2	TTCGGATCCTTAGAAACTAAATGTGGTTCTC
	alr2231_FW2	TACTAAACTGCGGGGAGAAG
	alr2231_RV2	AATTAACTGCAGAAGCTTGAAACTAAAAGTAGTAC
	all4499_FW2	GGGCATTCCAAGCAC
	all4499_RV2	CTCAGCACCAGTAGC
	alr4550_FW2	CCGCTACAGTAACGAAG
	alr4550_RV2	CAATGCGGTAGATGGG
	alr4741_FW2	GGCGGAAGATATAGTC
	alr4741_RV2	CCAAGCTAATCTCGCC
	all5191_FW2	CCCATGATAGTTGGGC
	all5191_RV2	GGAAATTGAACCGTCTCC
	all7614_FW2	CAGCCATCACAAAC
	all7614_RV2	GCTGCATAGTTACCATCG
	alr0075_FW	ATGGATACGCCCAATATGGT
	alr0075_RV	TCCCAAGTCACCACGAAATA
	alr2269_FW	AGCCCAACCCCGTCTTA
	alr2269_RV	AGGCTGTCCATTCTGCTCTT
	alr4893_FW	ATCGGCAAACCCATCAG

	alr4893_RV	CCACGGACGGAGTTAGAA
qRT-PCR porins	0834-qRT-Fw	CGCGATTAGAACTTGTCTCC
	0834-qRT-Rw	GCTAGCGAAGAACAGGTTAC
	2231-qRT-Fw	TACTAAACTGCGGGGAGAAG
	2231-qRT-Rw	CCTCAAGATTCGGTCTGCTA
	4499-qRT-Fw	CAGGATTAGCCGCACTATTC
	4499-qRT-Rw	TATCTACCGCCAAGCCTATG
	4550-qRT-Fw	CGCGTATCTGACAACATC
	4550-qRT-Rw	GAAGGTGGTTCTCAGAGT
	4741-qRT-Fw	TGCTGATATCCAGGCTCAAC
	4741-qRT-Rw	CTCCATCACCTGGACGATTC
	5191-qRT-Fw	ATCGCGCCATAGGGACTAGC
	5191-qRT-Rw	GGTGCAGGTATCGGGATTTC
	7614-qRT-Fw	GCTGCATAGTTACCATCG
	7614-qRT-Rw	GTCCCATTTGGTAGTGGTAG
	rnpB-qRT-F1	GTAGGCGTTGGCGGTTG
	rnpB-qRT-R1	CACTGGACGTTATCCAGC

Table S2. Plasmids used in this study.

Plasmid Name	Marker	Properties	Reference
pCSV3	Sp^R Sm^R		[33]
pCSV3-*alr0834*	Sp^R Sm^R	pCSV3 with fragment of *alr0834*	
pCSV3-*alr2231*	Sp^R Sm^R	pCSV3 with fragment of *alr2231*	
pCSV3-*all4499*	Sp^R Sm^R	pCSV3 with fragment of *all4499*	
pCSV3-*alr4550*	Sp^R Sm^R	pCSV3 with fragment of *alr4550*	In here
pCSV3-*alr4741*	Sp^R Sm^R	pCSV3 with fragment of *alr4741*	
pCSV3-*all5191*	Sp^R Sm^R	pCSV3 with fragment of *all5191*	
pCSV3-*all7614*	Sp^R Sm^R	pCSV3 with fragment of *all7614*	

Table S3. *Anabaena* strains used in this study and their respective genotype

Name	Genotype	Segregated	Reference
Anabaena sp. PCC 7120	-	-	
AFS-I-*alr0075*	*alr0075*::CSV3	no	[56]
AFS-I-*alr2269*	*alr2269*::CSV3	no	[56]
AFS-I-*alr4893*	*alr4893*::CSV3	no	[56]
AFS-I-*alr0114*	*alr0114*::CSV3	no	[44]
AFS-I-*alr0834*	*alr0834*::CSV3	yes	In here
AFS-I-*alr2231*	*alr2231*::CSV3	no	In here
AFS-I-*all4499*	*all4499*::CSV3	no	In here
AFS-I-*alr4550*	*alr4550*::CSV3	yes	In here
AFS-I-*alr4741*	*alr4741*::CSV3	yes	In here
AFS-I-*all5191*	*all5191*::CSV3	yes	In here
AFS-I-*all7614*	*all7614*::CSV3	yes	In here
